# Anti-EGFR therapy in metastatic colorectal cancer: mechanisms and potential regimens of drug resistance

**DOI:** 10.1093/gastro/goaa026

**Published:** 2020-06-23

**Authors:** Qing-Hai Li, Ying-Zhao Wang, Jian Tu, Chu-Wei Liu, Yu-Jie Yuan, Run Lin, Wei-Ling He, Shi-Rong Cai, Yu-Long He, Jin-Ning Ye

**Affiliations:** g1 Department of Gastrointestinal Surgery, the First Affiliated Hospital of Sun Yat-sen University, Guangzhou, Guangdong, P. R. China; g2 Department of Musculoskeletal Oncology, the First Affiliated Hospital of Sun Yat-sen University, Guangzhou, Guangdong, P. R. China; g3 Department of Radiology, the First Affiliated Hospital of Sun Yat-sen University, Guangzhou, Guangdong, P. R. China

**Keywords:** metastatic colorectal cancer, EGFR, drug resistance, cetuximab, panitumumab

## Abstract

Cetuximab and panitumumab, as the highly effective antibodies targeting epidermal growth factor receptor (EGFR), have clinical activity in the patients with metastatic colorectal cancer (mCRC). These agents have good curative efficacy, but drug resistance also exists at the same time. The effects of *KRAS*, *NRAS*, and *BRAF* mutations and *HER2* amplification on the treatment of refractory mCRC have been elucidated and the corresponding countermeasures have been put forward. However, the changes in EGFR and its ligands, the mutations or amplifications of *PIK3CA*, *PTEN*, *TP53*, *MET*, *HER3*, *IRS2*, *FGFR1*, and *MAP2K1*, the overexpression of insulin growth factor-1, the low expression of Bcl-2-interacting mediator of cell death, mismatch repair-deficient, and epigenetic instability may also lead to drug resistance in mCRC. Although the emergence of drug resistance has genetic or epigenetic heterogeneity, most of these molecular changes relating to it are focused on the key signaling pathways, such as the RAS/RAF/mitogen-activated protein kinase or phosphatidylinositol 3-kinase/Akt/mammalian target of the rapamycin pathway. Accordingly, numerous efforts to target these signaling pathways and develop the novel therapeutic regimens have been carried out. Herein, we have reviewed the underlying mechanisms of the resistance to anti-EGFR therapy and the possible implications in clinical practice.

## Introduction

Colorectal cancer (CRC) is one of the most common tumors. Data from 185 countries have shown that >1.8 million new CRC cases and 881,000 deaths were estimated to have occurred in 2018 and the incidence and mortality rank third and second, respectively [1]. In addition, ∼20% of new cases are diagnosed as metastatic colorectal cancer (mCRC) at the time of initial visiting [2]. Fortunately, although the incidence and mortality of CRC in some underdeveloped areas are still increasing, they are declining in the whole world, especially in the developed countries [3]. At present, a limited number of targeted agents such as cetuximab, panitumumab, bevacizumab, aflibercept, and regorafenib have been shown to be active in the treatment of the patients with mCRC (hereinafter referred to as the patients or mCRCs). However, a number of the patients benefit a lot from the clinical application of these drugs [4–8].

Cetuximab is a chimeric (mouse/human) IgG-1 monoclonal antibody (mAb). As a targeted drug, cetuximab can competitively bind to epidermal growth factor receptor (EGFR) with the natural ligands such as EGF [9]. Its main pharmacological mechanism is to inhibit the phosphorylation of EGFR tyrosine kinase caused by the ligands binding and then block a series of reactions such as gene transcription and cell proliferation induced by the activation of the phosphatidylinositol 3-kinase (PI3K)/Akt/mammalian target of rapamycin (mTOR) pathway and the RAS/RAF/mitogen-activated protein kinase (MAPK) pathway. Cetuximab can also mediate antibody-dependent cellular cytotoxicity and receptor internalization, so as to fully play the antitumor role [10–12]. The major side effects of cetuximab are skin toxicity and venous thromboembolic; skin rash may be related to the prognosis [13–17]. Panitumumab is a recombinant humanized IgG-2kappa mAb; both its pharmacological mechanisms and side effects are similar to those of cetuximab. In September 2006, panitumumab was approved by the US Food and Drug Administration as a single agent for the treatment of advanced CRC, but now it is mainly used in the combined-therapy regimens with other chemotherapeutic agents.

Although the use of cetuximab and panitumumab has greatly improved the overall survival (OS) and other clinical outcomes of mCRCs [4, 5], some patients cannot benefit from it. The discovery of the relationship between *KRAS* gene mutations and resistance to anti-EGFR mAbs has aroused the interest of a large number of scholars in the study of drug resistance. In this review, the molecular mechanisms of the resistance to anti-EGFR mAbs and the potential therapy regimens will be systematically elaborated on from three aspects: abnormal molecules in the EGFR pathway, abnormal activations between the paralleled pathways, and the other mechanisms.

## Abnormal molecules in the EGFR pathway

### Abnormalities of EGFR and its ligands

EGFR is one of the members of the ERBB/HER protein family that is composed of EGFR (HER1/ERBB1), HER2 (ERBB2/neu), HER3 (ERBB3), and HER4 (ERBB4). It consists of extracellular domains, transmembrane domains, and signal-transduction domains with tyrosine kinase activity. After binding to its ligands through the extracellular binding region, the proteins in the signal-transduction region phosphorylate and this finally leads to the intracellular cascade reactions that mainly promote cell proliferation [10, 18, 19]. In principle, the mutations of *EGFR*, the low expression of EGFR, and the changes in its ligands are detrimental to the efficacy of anti-EGFR therapy in some cancers. For example, in lung cancer, the *EGFR* gene copy number (GCN) or mutations (especially *EGFR*^T790M^) has been confirmed to be related to the resistance of molecule-targeted drugs [20, 21]; in the latest molecular-detection guideline of lung cancer, the importance of both *EGFR* GCN and mutations detection before the targeted treatment is reiterated [22]. Although some studies have pointed out that patients are all responsive to anti-EGFR mAbs regardless of the status of the EGFR [23–25], emerging evidence has shown that *EGFR* GCN and the acquired mutations of extracellular domains are related to the resistance to anti-EGFR mAbs in mCRCs [26–31], but EGFR-related detection in mCRCs has not been recommended at present [32]. Therefore, whether this indicator can be used as an efficacy predictor of anti-EGFR therapy in patients should be further investigated.

**Figure 1.**
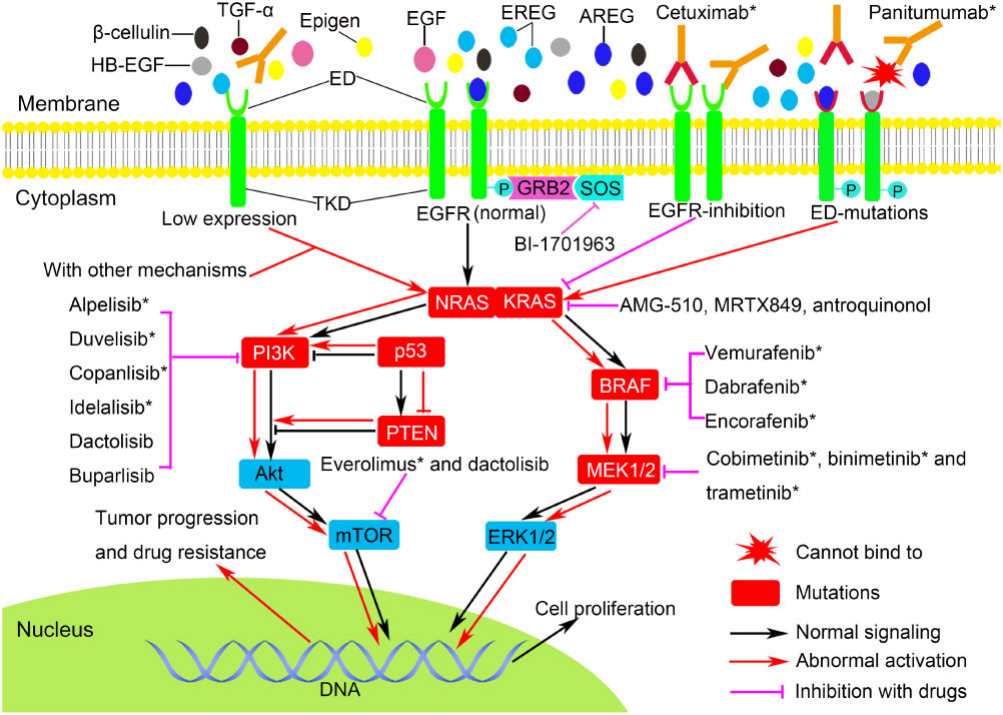
EGFR signaling and potential regimens in cetuximab- or panitumumab-resistant mCRC. In addition to NRAS/KRAS/BRAF mutations, low expression of EGFR/AREG/EREG, the mutations of extracellular domains of EGFR, PIK3CA, PTEN, TP53, and MEK1 are related to cetuximab or panitumumab resistance. The agents targeting BRAF and MEK1/2 have been approved for the subsequent therapy of advanced or metastatic CRC; some other targeted drugs such as PI3K, Mtor, and RAS inhibitors also deserve our attention, especially the KRAS G12C inhibitors AMG-510 and MRTX849. EGF, epidermal growth factor; EGFR, epidermal growth factor receptor; HB-EGF, heparin-binding EGF; TGF-α, transforming growth factor α; AREG, amphiregulin; EREG, epiregulin; ED, extracellular domains; TKD, tyrosine kinase domains; GRB2, growth factor receptor bound-2; SOS, son of sevenless; MEK1/2, mitogen-activated protein kinase kinase; ERK1/2, extracellular-signal-regulated kinase; PI3K, phosphatidylinositol 3-kinase; mTOR, mammalian target of rapamycin; p, phosphorylation; * have been approved for markets.

The ligands of EGFR mainly include EGF, transforming growth factor α (TGF-α), amphiregulin (AREG), epiregulin (EREG), epigen (EPGN), β-cellulin, and heparin-binding EGF (HB-EGF). As early as 2007, researchers had noticed that the expression levels of EREG and AREG are related to the efficacy of cetuximab in mCRCs [33]. Subsequently, accumulating studies have confirmed this finding; they have found that the higher the expression levels of these ligands, the better the observed efficacy of anti-EGFR mAbs in mCRCs can be, but this correlation is not consistent in patients with *KRAS* mutations [34]. In 2016, a randomized clinical trial of panitumumab plus irinotecan and ciclosporin in the treatment of advanced CRC has come out with a new ligand-expression model and pointed out that the expression levels of EREG and AREG are related to the therapeutic efficacy of panitumumab [35]. All of the above studies have suggested that the downregulation of EGFR ligands, especially EREG and AREG, may be one of the mechanisms of resistance to anti-EGFR mAbs. Therefore, the expression levels of EREG and AREG may be valuable markers for the choice of regimens in mCRCs, and it is worthy to be further optimized and then used for clinical guidance in order to reduce drug resistance and improve anti-EGFR mAbs efficacy (Figure 1).

### Mutations of *RAS*

*KRAS*, *HRAS*, and *NRAS* are three major members of the RAS family. *RAS* as a proto-oncogene encodes the protein with GTPase activity, which plays a role of transducing and self-inactivating the signals in EGFR signaling. At present, the abnormalities of the *RAS* family have been reported to be related to a variety of tumors, and the mutations of *KRAS* and *NRAS* closely relate to CRC among them, but the relationship between CRC and the abnormality of *HRAS* is rarely reported [36]. According to the statistics, ∼37%–45% of CRCs harbor *KRAS* mutations in exon 2 (codons 12 or 13) and nearly 10% of CRCs harbor non-*KRAS* exon 2 *RAS* mutations that consist of non-exon 2 *KRAS* mutations (codons 59 or 61 mutations in exon 3, codons 147 or 117 mutations in exon 4) and *NRAS* mutations (codons 12 or 13 in exon 2 and codons 59 or 61 in exon 3) [5, 37–42].

Although studies have shown that the status of *KRAS* and *NRAS* is independent of the tumor staging [37, 43], since Lievre *et al.* [44] first reported that the mutations of *KRAS* can reduce the efficacy of anti-EGFR mAbs in 2006, emerging studies have confirmed that *RAS* mutations are the major causes of resistance to anti-EGFR therapy [5, 43, 45–50]. These pieces of evidence are mainly as follows: not only the resistance to anti-EGFR mAbs is related to *KRAS* mutations in exon 2 codons 12, but also the other mutant types of *RAS* such as codons 13 mutation in exon 2 and non-*KRAS* exon 2 *RAS* mutations can render the resistance to cetuximab or panitumumab in mCRCs; in addition to primary *RAS* mutations, there are many patients who harbor acquired *RAS* mutations in response to EGFR blockade. At present, the genotypes of *KRAS* and *NRAS* have become the main basis for choosing the chemotherapy regimen in mCRCs; anti-EGFR therapy in patients with *KRAS* and *NRAS* wild type (WT) has greatly improved their benefits. At the same time, in order to solve the problem of genes-detection sensitivity and specificity, not only should genes testing be performed only in the laboratories that are certified under the Clinical Laboratory Improvement Amendments of 1988 (CLIA-88), but also the latest National Comprehensive Cancer Network (NCCN) Colon/Rectal Cancer Panel and tumor-molecular-detection guidelines have recommended the next-generation sequencing (NGS) technology (Table 1) [32, 51].

**Table 1.** Currently recommended molecular testing for colorectal cancer

Biomarker	Test defect	Time	Technology	Recommendations	Evidence
KRAS/NRAS^a^	Mutation	Workup for metastatic disease (suspected or proven)	NGS^b^, PCR	Avoid cetuximab or panitumumab treatment in mCRC patients who have tumors with *KRAS* and *NRAS* mutations (exons 2, 3, and 4 in both)	NCCN indicates lower-level, uniform acceptance (category 2A); but many believe classification is high-level, uniform acceptance
BRAF^a^	Mutation V600E	Workup for metastatic disease (suspected or proven)	NGS, PCR^b^	Cetucimab or panitumumab treatment is not recommended in mCRC patients harboring *BRAF^V600E^* mutation unless given with *BRAF* inhibitors with or without MEK inhibitors at the same time	NCCN indicates lower-level, uniform acceptance (category 2A); but many believe classification is high-level, uniform acceptance
HER2	Amplification	Workup for metastatic or advanced disease (suspected or proven)	NGS, IHC, or FISH, need more evidence	Patients with *HER2* amplification may be resistance to Cetuximab or panitumumab, trastuzumab plus (pertuzumab or lapatinib) regimen is an option for mCRC patients with *HER2* amplified and *RAS* WT, but need more evidence^d^	NCCN indicates lower-level, wide acceptance (category 2B); but many believe classification is high-level, wide acceptance
NTRK	Gene fusion	Workup for metastatic disease (suspected or proven)	NGS, FISH, PCR	Larotrectinib is a treatment option for mCRC patients who are *NTRK* gene-fusion positive	NCCN indicates lower-level, uniform acceptance (category 2A); but many believe classification is high-level, uniform acceptance
MMR/MS	dMMR^c^/MSI-H	All patients with colon or rectal cancer	NGS, IHC^c^, PCR	Stage II MSI-H CRC patients may have a good prognosis but lack of efficacy of fluorouracil-based adjuvant therapy (nivolumab with or without ipilimumab) or pembrolizumab regimens are recommended for the patients with dMMR/MSI-H only	NCCN indicates lower-level, uniform acceptance (category 2A); but many believe classification is high-level, uniform acceptance

NGS, next-generation sequencing; PCR, polymerase chain reaction; NCCN, National Comprehensive Cancer Network; IHC, immunohistochemistry; MMR, mismatch repair; dMMR, mismatch repair-deficient; MS, microsatellite; MSI-H, microsatellite highly instability; mCRC, metastatic colorectal cancer; FISH, fluorescence *in situ* hybridization; WT, wild-type.

^a^ *KRAS* and *NRAS* are determined alongside *BRAF* mutations.

^b^ Testing can be performed on primary and/or metastatic colorectal tissue specimens.

^c^ IHC refers to staining tumor tissue for protein expression of the four MMR genes known to be mutated in Lynch syndrome (*MLH1*, *MSH2*, *MSH6*, and *PMS2*).

^d^ If no previous treatment with HER2 inhibitor.

The structures of *KRAS*- and *NRAS*-mutant proteins are complex; although they have been known for a long time, the research and development of the related agents such as molecule-targeted drugs, mAbs, or vaccines are still quite difficult, and there is no RAS-targeted drug that has been successfully marketed so far. Therefore, the chemotherapy regimens with bevacizumab are still recommended in the guidelines for patients harboring *RAS* mutations. However, there have been several agents that have deserved our attention in recent years. In 2013, a paper showed that KRAS G12C could be targeted by a covalent compound that locks the mutant protein in its inactive GDP-bound state, which is supported by the discovery that KRAS G12C retains the highest residual intrinsic GTPase activity [52]. In addition, after the development and verification of the tool covalent compounds ARS-853 and ARS-1620, the first specific irreversible covalent inhibitors of KRAS-G12C in the clinic came from Amgen, AMG510, then from Mirati Therapeutics, MRTX849 (Figure 1). These two agents have been studying in clinic trials or *in vitro* and have been showing excellent antitumor efficacy that includes driving antitumor immunity [53–55]. Some clinic trials still aim to further confirm this evidence (NCT03600883 and NCT03785249). BI-1701963 is a non-specific inhibitor of KRAS; it blocks RAS signal transduction through inhibiting SOS1 selectively. The preclinical study has proved that BI-1701963 is effective for *KRAS*-mutant tumors. At present, China and other countries are participating in the global early clinical trial of this drug (NCT04111458). Antroquinol is also a non-specific inhibitor of RAS; it can indirectly inhibit the activation of RAS and RAS-related GTP-binding protein by inhibiting the activity of isoprenyl transferase and then promoting apoptosis. Preclinical studies have proved that antroquinol has a curative effect on *RAS*-mutant CRC [56, 57]. The trial of antroquinol as a first-line treatment of metastatic pancreatic cancer has been started (NCT03310632), but the research on mCRCs needs to be carried out. In addition, the vaccines designed for *RAS*-mutant peptide or immunotherapy with polyclonal T-cells for the patients with *RAS* mutations could also be a great breakthrough [58–61].

### Mutations of *BRAF*

*BRAF* proto-oncogene belongs to the *RAF* genes family that includes two other members: *CRAF* and *ARAF*. As an important component of the RAS/RAF/MAPK pathway, BRAF mediates the combination of RAF and MAPK kinase (MAPKK/MEK1/2) in the signal transduction and regulates cell proliferation [62]. Statistics have shown that 30%–60% of melanoma, 30%–50% of thyroid cancer, and 5%–9% of CRC harbor *BRAF* mutations. The features of *BRAF* mutations in CRC are likely to occur in smokers and elderly and female patients whose primary tumor is mainly located in the right colon. Such patients commonly have high-grade cancers at diagnosis and a higher number of cancer-involved lymph nodes; they are more likely to be microsatellite instability-high (MSI-H) or mismatch repair-deficient (dMMR) and to progress rapidly [63].

*BRAF* ^V600^ mutations are the most common mutations (about 80%). Among them, *BRAF*^V600E^ accounts for >90% of BRAF^V600^ mutations, which refers to the T-to-A changing at nucleotide 1799 and a substitution of valine to glutamic acid [64]. The result of these changes is the rendering of BRAF protein to be an active monomer that is independent of the upstream signals, causing the synchronous and continuous activation of downstream pathways and promoting cell proliferation and differentiation. Some other rare *BRAF*-mutant types include mutant-mutant BRAF dimers that lead to high extracellular-signal-regulated kinase (ERK1/2) activation in a RAS-independent manner and mutant *BRAF*-wild-type CRAF dimers that amplify the signaling downstream of RAS. Many studies have proved that patients harboring *BRAF* mutations are resistant to cetuximab or panitumumab and have a generally poor prognosis [4, 5, 41, 65–67]. Therefore, *BRAF* status is a strong predictor for the predictive prognosis of CRC. In addition to *RAS* typing, high-sensitivity typing of the *BRAF* gene in mCRCs is equally important (Table 1) [32].

Different from the development of RAS inhibitors, many breakthroughs have been made in the development of BRAF inhibitors. At present, the approved BRAF monomers-targeted inhibitors include vemurafenib, dabrafenib, and encorafenib (LGX818) (Figure 1). They are mainly used in the treatment of melanoma, but the efficacy of single-agent therapy for mCRCs remains unclear [68], which is mainly related to the fact that, after inhibiting the MAPK pathway using BRAF inhibitors, EGFR can be activated by feedback in mCRCs, whereas melanoma only expresses low-level EGFR [69, 70]. However, the emergence of BRAF inhibitors makes the combined-therapy regimens possible. According to the latest NCCN Guidelines of Colon/Rectal Version, for the patients with *BRAF*^V600E^ mutation, the first-line targeted-therapy regimens should include bevacizumab, while the subsequent options include targeting BRAF, MEK, and EGFR at the same time, such as dabrafenib plus trametinib and cetuximab or panitumumab, or encorafenib plus binimetinib (MEK162) and cetuximab or panitumumab. These two regimens have been proven to be effective and tolerable, which improves the benefits of the patients to some extent [71–73]; another multi-targeted-therapy regimen [MEK162 plus LGX818 and LEE011 (cyclin-dependent kinase (CDK) 4/CDK6 inhibitor)] is in a phase IB/II clinical trial (NCT01543698). In addition, as mentioned above, this type of patient situation is often accompanied by dMMR/MSI-H, so that anti-programmed death1 (PD-1)/anti-programmed death ligand 1 (PD-L1) and anti-cytotoxic T-lymphocyte antigen 4 (CTLA-4) immunotherapy may also have an ideal therapeutic effect [63, 74].

### Mutations of *PIK3CA*, *PTEN*, and *TP53*

The *KRAS*/*NRAS*/*BRAF* status is a strong predictor of anti-EGFR mAbs efficacy in mCRCs, but more predictive factors are needed for the better selection of patients who will benefit highly from anti-EGFR mAbs. *PIK3CA* encodes p110alpha, a catalytic subunit of PI3K that mediates the PI3K/AKT pathway and promotes cell survival, whereas *PTEN* and *TP53* are negative regulators of this pathway as the tumor-suppressor genes. PTEN plays a role as 3-phosphatase that dephosphorylates PIP3 or PIP2, and therefore inhibits the activation of PI3K and blocks this pathway [18, 75, 76]. *TP53* encodes p53 protein, which acts not only as a guardian of the genome in normal cells, but also as an inhibitor of the oncogenes in cancer cells. Therefore, changes in *PIK3CA* could lead to the abnormal activation of the PI3K pathway, while the abnormalities of *PTEN* and *TP53* genes could lead to the release of pathway inhibition. The final results could be that the regulation of the cells is out of control and abnormal proliferation of the cells occurs. The statistics have shown that the mutation rates of *PIK3CA* and *PTEN* in CRCs are ∼12% and ∼6%, respectively; mutations of *PIK3CA* are common in exon 9 and exon 20, and mainly occur in colon-cancer patients [77–79]. However, the frequency of *TP53* mutations in CRCs is still unclear.

Increasing evidence has shown that the efficacy of anti-EGFR therapy will be greatly reduced in patients harboring the above mutations or expressing low PTEN and TP53 levels [80–87]. Although the differences in the clinical outcomes between patients with single exon 9 mutations of *PIK3CA* and patients with single exon 20 mutations of *PIK3CA* are not significant, when there are double mutations in patients, the clinical outcomes will be worse [85, 86]. Therefore, although there is no guideline to recommend using the genotypes of *PIK3CA*, *PTEN*, and *TP53* to guide the choice of treatment regimens in CRCs, the role of these three genotypes or the expression levels of PTEN and p53 in anti-EGFR mAbs resistance should not be ignored.

The PI3K pathway has two major targets: PI3K and mTOR (Figure 1). At present, mTOR inhibitors such as everolimus are mainly used in the targeted therapy of advanced renal-cell carcinoma; they have not been recommended for the treatment of mCRC [88, 89]. Although they have shown curative efficacy in mCRCs, their safety and effectiveness still need to be further studied. At the same time, the study and development of PI3K inhibitors have achieved preliminary success: a number of PI3K inhibitors such as alpelisib (BYL719), duvelisib, copanlisib, and idelalisib have been approved for the targeted therapy of *PIK3CA*-mutant head and neck tumors, breast cancer, or lymphoma; besides, dactolisib (BEZ235, dual PI3K/mTOR inhibitor), PI-103, buparlisib (BKM120), and so on have been approved for the clinical trials of solid tumors. However, similarly to mTOR inhibitors, there are only a few studies that relate to mCRCs. A phase Ib dose-escalation study has shown that the combined treatment of encorafenib plus cetuximab and alpelisib is tolerable and provides promising clinical activity in the difficult-to-treat patient population with *BRAF*-mutant mCRC [90]. The results of some ongoing clinical trials that aim to combine PI3K or mTOR inhibitors with other targeted agents (NCT01719380, NCT01337765, and NCT01591421) to block the PI3K and MAPK pathways at the same time and eliminate drug resistance in mCRCs deserve our exploration. In addition, because *PTEN* mutations are also often accompanied by MSI-H [79], researchers are trying to use one PI3K inhibitor in combination with nivolumab to treat patients (NCT03711058, NCT03735628).

## Abnormal activations between the parallel pathways

### Amplifications or mutations of *HER2* and *HER3*

HER2 and HER3 are two distinct members of the ERBB/HER protein family; they are both non-autonomous. HER2 (ERBB2/neu) does not bind with EGF-like ligands, but it acts as the preferred heterodimer partner of the other three members of the ERBB family [91]. Heterodimers such as EGFR/HER2 have higher affinity and specificity for the ligands [92]. Meanwhile, HER2 can bind more phosphotyrosine-binding protein than the other receptors [93]. In addition, the internalization process of dimers-containing HER2 will be slower and the receptors will return to the cell surface more effectively to receive the second stimulation [94, 95]. While the kinase activity of HER3 (ERBB3) is defective, it works by forming heterodimeric complexes with other receptors that are capable of generating potent cellular signals and then recruiting PI3K to six distinct sites in heterodimeric complexes, thus strongly activating PI3K in a RAS-independent manner [96]. Therefore, both *HER2* and *HER3* amplifications (also HER2 and HER3 overexpression) and mutations could increase the malignancy degree of the tumors through these mechanisms.

Although the amplification and mutations of *HER2* are most common in breast cancer, research over the past decade has shown that 3%–5% of CRCs harbor primary overexpression of HER2 or *HER2* mutations, and the prevalence is higher in *RAS* and *BRAF* WT CRCs (reported in about 5%–14%) (according to HERACLES criteria: immunohistochemistry 3+ or 2+ in >50% cells confirmed by fluorescence *in situ* hybridization) [97–100]. Meanwhile, 2% of patients harbor acquired mutations or amplification of *HER2* gene after receiving anti-EGFR therapy, and their primary tumors mostly focus in the left colon and rectum and are *RAS* WT [101, 102]. However, the prevalence of *HER3* amplification and mutations seems higher than that of *HER2*. According to the statistics, 5.7%–11% of CRCs harbor *HER3* mutations [100, 103] and 51%–70% of CRCs show the overexpression of HER3 protein, but the evaluated criteria of HER3 overexpression are different [104, 105]. Since 2011, emerging studies have reported that the resistance to anti-EGFR mAbs is related to the primary or acquired amplification and mutations of *HER2* [106–110], but the evidence does not support a prognostic role for HER2 overexpression [111]. As for HER3, although the study of the relationship between HER3 and anti-EGFR mAbs resistance is relatively later and smaller than that of HER2, much evidence has also revealed that abnormalities in HER3 could also reduce the efficacy of anti-EGFR therapy in the treatment of mCRCs [104, 112–114]. These findings reveal a novel mechanism of resistance to anti-EGFR mAbs: the abnormalities of HER2 and HER3. Therefore, HER2 has become a new target of mCRC genotyping and treatment up to now (Table 1). However, the diagnostic criteria for the overexpression of HER3, more authoritative evidence of the relationship between HER3 and the resistance to anti-EGFR mAbs, and HER3-targeted agents approved for markets are all required to render HER3 as a new genotyping and treatment target of mCRC.

HER2-targeted agents that have been approved for markets include the mAbs trastuzumab and pertuzumab, the inhibitors of ERBB family tyrosine kinase neratinib, afatinib, and pyrotinib, the conjugated agents trastuzumab-emtansine (T-DM1) and tratuzumab-hyaluronidase-oysk, and the dimerization inhibitors of EGFR/HER2 tyrosine kinase lapatinib (Table 2); they are mainly approved for the treatment of breast cancer. A number of multicenter, open phase II clinical trials have shown that trastuzumab plus lapatinib has good efficacy and tolerability in the treatment of patients who are HER2-positive [98, 115]; therefore, the latest NCCN guideline recommends that trastuzumab plus pertuzumab or lapatinib can be used in *RAS* WT mCRCs with *HER2* amplification for the subsequent therapy. Although a randomized, open-label phase II trial of afatinib vs cetuximab in mCRCs has shown that the efficacy of afatinib is modest in patients with *KRAS* mutations [116], the use of tyrosine kinase inhibitors in mCRCs with or without *HER2* mutations or amplification needs more evidence to support it [108]. The conjugated agents ado-trastuzumab-emtansine (T-DM1) and tratuzumab-hyaluronidase-oysk were approved for markets by the USA, Europe, and China for the treatment of HER2-positive breast cancer in 2019, but they have been rarely tried for mCRC. At present, many related clinical trials have been started (NCT03418558, NCT03225937, NCT03457896, NCT01919879, and NCT03843749). However, no HER3-targeted agent has been approved for markets. HER3-specific or bispecific inhibitors that have been approved for clinical trials in the past 5 years mainly include the following types: the humanized HER3 mAbs lumretuzumab, ISU104, and CDX-3379; the dual-action HER3/EGFR mAbs zenocutuzumab, duligotazumab (MEHD7945A); and the novel conjugated agent U3-1402 (Table 2); however, the efficacy of these agents in the trials is inconsistent and controversial [117–121]. In addition, both the efficacy and the tolerability of HER2/HER3-targeted drugs plus EGFR mAbs are also worthy of exploration.

**Table 2.** Abnormal activations between the parallel pathways and the potential regimens

Receptor/ligand	Functions	Change	Main targeted drugs (for market* or clinic trial)
HER2	As the preferred heterodimeric partner of the other three ERBB members and amplifying the cascades	Amplification or mutations	HER2 antibodies: trastuzumab*, pertuzumab Kinase inhibitors: neratinib*, afatinib*, pyrotinib*; Specific kinase inhibitors: lapatinib; tucatinib Conjugated drugs: ado-trastuzumab-emtansine*, tratuzumab/hyaluronidase-oysk*
HER3	As the heterodimeric partner of the growth factor receptors and directly activating PI3K signaling in a RAS-independent way	Amplification or mutations	HER3 antibodies: lumretuzumab, ISU104, CDX-3379 Dual HER3/EGFR antibodies: zenocutuzumab, duligotazumab (MEHD7945A) Kinase inhibitors: neratinib*, afatinib*, pyrotinib* Conjugated drugs: U3-1402
HGFR	Binding to HGF and activating multiple signal-transduction pathways, such as the MAPK pathway, the PI3K pathway, the NF-κB pathway, and the STAT3 pathway	Amplification	Dual EGFR/HGFR antibodies: Sym-015, JNJ-61186327, LY3164530, EMB01 Kinase inhibitors: crizotinib*, cabozantinib* Specific inhibitors: volitinib Conjugated drugs: ABBV-399, SHR-A1403
IGF1	Binding to IGF1R and activating multiple growth-related signal-transduction pathways that include the MAPK pathway and the PI3K pathway	Amplification	IGF1R antibodies: temprotumumab*, ganitumab, dalotuzumab, IMC-A12, dalotuzumab Dual IGFR/IR tyrosine kinase inhibitor: linsitinib
IGF2	Similar to IGF1	Amplification	

HGF, hepatocyte growth factor; HGFR, hepatocyte growth factor receptor; PI3K, phosphatidylinositol 3-kinase; MAPK, mitogen-activated protein kinase; STAT3, transducer and activator of transcription 3; IGF, insulin-like growth factor; IGF1R, insulin-like growth factor 1 receptor; IR, insulin receptor; *have been approved for markets.

### Amplification of *MET*

Hepatocyte growth factor receptor (HGFR, also known as MET tyrosine kinase receptor) is encoded by proto-oncogene *MET* [122], whereas its ligand HGF is produced by the surrounding fibroblasts and plays a biological effect by paracrine on the adjacent cells that express HGFR [19, 123]. The binding of HGF and HGFR leads to efficient activation of downstream signal-transduction pathways that include MAPK cascades, PI3K/Akt axis, the nuclear factor-κB inhibitor-α (IκBα)–nuclear factor-κB (NF-κB) complex, and the signal transducer and activator of transcription 3 (STAT3) [124]; besides, HGFR can also be coupled with HER3 to form a heterodimer under certain conditions [125]. Therefore, *MET* can not only participate in the occurrence of various morphological events both in the embryo and adulthood, but also drive the malignant progress or drug resistance of a variety of tumors, including CRC.

Currently, both preclinical and clinical studies have shown that *MET* abnormalities in CRC mainly manifest in gene amplification that can resist the inhibition effect of anti-EGFR mAbs on tumors and rarely in mutations [126–128]. Although the incidence of innate *MET* amplification in CRC patients is only 2%–4% [129, 130], as *MET* is similar to *HER2*, some CRCs harbor acquired *MET* amplification during the process of anti-EGFR therapy [128, 131, 132]. Hence, the failure of anti-EGFR therapy caused by the amplification of *MET* cannot be ignored.

At present, some non-specific inhibitors of MET tyrosine kinase such as crizotinib and cabozantinib have been approved for the treatment of other cancers, but all of the macromolecule or specific agents targeting HGFR/HGF are still at the clinical-trials stage. In recent years, the agents and relative clinical trials that deserve our special attention mainly include specific MET tyrosine kinase volitinib, conjugated drugs ABBV-399 and SHR-A1403, and dual-action EGFR/HGFR mAbs Sym-015, JNJ-61186372, LY3164530, and EMB01 (Table 2). Before that, many trials were related to the receptor mAb onartuzumab, the ligand mAb rilotumumab (AMG102), and the selective non-ATP competitive kinase inhibitor tivantinib (ARQ197). Both phase I and phase II clinical trials of those drugs have also shown great efficacy and tolerability in mCRCs [131, 133–135]. However, with many failures of those drugs in phase III clinical trials of other cancers [136–138], these drugs have not been approved for CRC yet in phase III clinical trials.

### Overexpression of IGF1 and IGF2

Insulin-like growth factor 1 and 2 (IGF1 and IGF2) are quite important growth factors in the human body. IGF1 receptor (IGF1R) is the common receptor of IGF1 and IGF2. After binding with them, IGF1R can activate multiple signal-transduction pathways, including the RAS/RAF/MAPK and PI3K/AKT pathway. Therefore, the IGF1R and EGFR pathways have a close crosstalk and participate in cell proliferation, differentiation, angiogenesis, apoptosis, and other life processes together [139, 140]. Although the concentration of IGF2 in blood circulation is higher than that of IGF1, IGF1 plays a more important role in tumor progression than IGF2 because it is regulated by growth hormones, is not easily degraded, and has 15 times higher affinity with IGF1R [141, 142].

The evidence from clinical trials has shown that the overexpression of IGF1 can render the obvious resistance to anti-EGFR mAbs in mCRCs [104, 143], whereas patients with IGF2 overexpression only show lower sensitivity and a marginal response to anti-EGFR therapy [144]. However, few studies have reported the relationship between abnormal IGF1R and cancers. An *in vitro* study has shown that the regimen of anti-EGFR mAb ICR62 plus IGF1R tyrosine kinase inhibitor NVP-AEW541 can synergistically enhance the antitumor effect in some CRC cells [145]. Consequently, IGF1 and IGF1R could be two potential factors for anti-EGFR mAbs resistance.

Since IGF1 and IGF2 both act on IGF1R to play a physiological role, many researchers are focusing on targeting IGF1R. However, there are few breakthroughs in the development of IGF1R-targeted agents. Although an IGF1R mAb temprotumumab has been approved for the treatment of thyroid-associated ophthalmopathy, there is no trial of this agent for cancers. A randomized phase II clinical trial showed that the IGF1R inhibitor IMC-A12 has not shown the expected efficacy *in vivo*, either alone or combined with cetuximab [146]. A larger-sample-size II/III clinical trial suggested that an IGF1R inhibitor dalotuzumab (MK-0646) plus irinotecan and cetuximab regimen has good tolerability in patients, but there is no significant improvement in OS [147]. The other IGF1R inhibitor ganitumab plus panitumumab also did not improve the OS of mCRCs in a phase IB/II clinical study [133]. A phase I clinical trial confirmed that a dual-action inhibitor linsitinib (OSI-906) that targets IGF1R and insulin receptor (IR) at the same time has good antitumor activity *in vivo* [148]; unfortunately, before reaching the maximum tolerated dose, the OSI-906 plus mTOR inhibitor everolimus regimen has not manifested clinical efficacy when being used to treat refractory CRC [149]. At present, although it is feasible to inhibit IGF1R in theory, more clinical evidence and novel targeted agents are needed to support that IGF1R can be a therapeutic target after the emergence of resistance to anti-EGFR mAbs.

## Other mechanisms

In addition to the primary or acquired changes in these common targets, some changes in genes or expression levels that have been rarely reported, unknown mutations, or epigenetic instability can also render the patients resistant to anti-EGFR therapy.

Some changes that have been rarely reported include *NTRK* gene fusion, *IRS2* amplification or mutations, *FGFR1* and *MAP2K1* mutations, and the low expression of the Bcl-2-interacting mediator of cell death (Bim) [28, 150, 151]; the occurrence of unknown mutations mostly relates to dMMR. Previous studies have suggested that dMMR is associated with a good prognosis in early-stage disease and is rare in advanced patients, especially in sporadic CRC [152]. However, the latest research has pointed out that, under the pressure of anti-EGFR drugs, human CRC cells with mismatch repair proficient/microsatellite stable (pMMR/MSS) can manifest dMMR phenotype in a variety of ways, including downregulating the expression of mismatch repair (MMR) genes (*MLH1*, *MLH2*, and *MLH6*), homologous recombination genes (*BBRCA2* and *RAD51*), and double-strand break repair gene *EXO1*, and gradually replacing high-fidelity DNA polymerase by low-fidelity DNA polymerases to participate in the DNA-replication process. Under the above integrated mechanisms, the genome of these cells begins to produce a large number of unknown adaptive mutations and MSI-H, which leads to these cells obtaining resistance to EGFR inhibitors. At the same time, the study also pointed out that the resistance to anti-EGFR agents in cancer cells will change from temporary to irreversible as the agents last longer [153], which put forward a new thinking on the choice of the targeted agents and the design of the treatment regimens.

Epigenetic instability includes aberrant DNA methylation, histone modification, chromosome remodeling, and non-coding RNA interference [154]; aberrant methylation phenotypes and microRNA (miRNA) interference are closely related to both the development and the drug resistance of CRC [155].

The data have shown that methylation events are more common than mutations in CRC patients [155, 156]. The whole-genome hypermethylation status of patients is significantly associated with the poor efficacy of anti-EGFR therapy, but many related mechanisms remain unclear [157]. At present, the study of cytosine-phosphate-guanine (CpG) island methylation phenotype (CIMP) is relatively extensive; however, due to lack of a unified definition standard, the prevalence of CIMP is temporarily unknown. Some analyses have shown that CIMP often accompanies *BRAF* mutations and relates to the low expression of MLH1, AREG, and EREG in CRCs, which may be a major mechanism by which patients are resistant to anti-EGFR mAbs [158, 159]. miRNA is a classification of endogenous small non-coding RNA that can regulate the expression of genes after transcription [160]. Although the study of miRNA interference with respect to resistance to anti-EGFR mAbs started relatively late, its role cannot be ignored due to the reversibility and activity of epigenetics that are similar to the interval drug resistance of mCRCs in clinic [161]. At present, miRNA-199a-5p/miRNA-375, miRNA-100/miRNA-125/miRNA-181a-5p, miRNA-425-5p, and miRNA-31-5p have been reported to be related to anti-EGFR therapy resistance in mCRCs [162–165] and the interference mechanisms of some miRNAs are relatively clear. MiRNA-199a-5p and miRNA-375 target PHLPP1, which indirectly activates the PI3K/AKT pathway [162], whereas miRNA-100/miRNA-125/miRNA-181a-5p regulates the Wnt/β-catenin-signaling pathway [163]. The above phenomena of epigenetic instability can interact with genetic mutations or amplification to mediate the resistance to anti-EGFR therapy in patients.

## Discussion and future perspective

**Figure 2.**
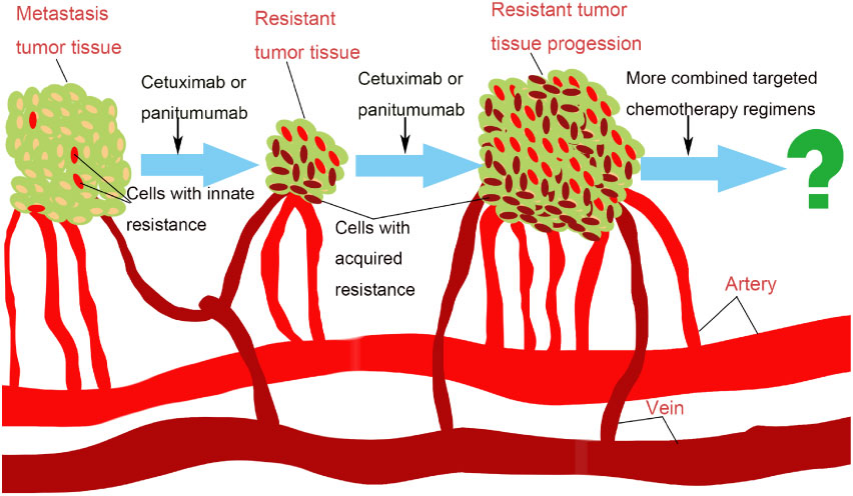
The clinical process of changing tumor inhibition to drug resistance and tumor progression. This process is extremely complex and highly heterogeneous because there are both innate and acquired factors that contribute to the occurrence of drug resistance and the strategy of combined targeted chemotherapy deserves our attention.

Anti-EGFR mAbs cetuximab and panitumumab were approved for the treatment of mCRC in 2004 and 2006, respectively; after years of clinical practice, they have been approved for the first-line treatment of *KRAS*/*NRAS*/*BRAF* WT mCRC. Although the OS, objective response rate, and progression-free survival of most patients with *KRAS*/*NRAS*/*BRAF* WT are significantly prolonged or increased after treatment with anti-EGFR mAbs, we have to face the reality that there are still some patients harboring the above genotypes who cannot obtain similar benefits, and even some patients who have experienced the clinical process of changing tumor inhibition to drug resistance and disease progression (Figure 2). This process is extremely complex and highly heterogeneous because there are both innate and acquired factors that contribute to the occurrence of drug resistance and, in addition to *KRAS*/*NRAS*/*BRAF* mutations, it is also related to a variety of molecular changes and genetic or epigenetic events. On the other hand, these mechanisms of drug resistance mainly manifest as EGFR-independent activations of the RAS/RAF/MAPK or PI3K/AKT pathway, which also provides some ideas for the solution to the anti-EGFR mAbs-resistance problem.

At present, the principle of precise classification for treatment is being widely adopted to solve this problem. The NCCN Guidelines of Colon/Rectal Cancer Version have recommended the treatment regimens of *KRAS*/*NRAS*/*BRAF* mutations, *HER2* amplifications, and dMMR/MSI (Table 1). Meanwhile, in order to obtain more accurate genotypes, the latest guidelines also have recommended using NGS technology for the genes detection of CRCs. However, with more agents that act on the resistance-related targets entering clinical trials or being approved for markets, the regimens of combined targeted chemotherapy could solve this problem under the premise of tolerable toxicity. Therefore, evaluation has now become the most important task (Figure 2), which should include the toxicity, the comparative efficacy, the best combination regimens, the timing of medication, and the appropriate efficacy evaluation biomarkers or index. We are looking forward to more positive results in order to improve the clinical efficacy of anti-EGFR therapy in mCRCs.

## Authors' contributions

All authors contributed to the concept development, determined the search strategy, evaluated the results for inclusion, and provided critical review of the manuscript. Finally, all authors have read and approved the final manuscript.
